# A comprehensive autoantigen-ome of autoimmune liver diseases identified from dermatan sulfate affinity enrichment of liver tissue proteins

**DOI:** 10.1186/s12865-019-0304-1

**Published:** 2019-06-26

**Authors:** Wei Zhang, Jung-hyun Rho, Michael H. Roehrl, Julia Y. Wang

**Affiliations:** 1 Department of Gastroenterology, Affiliated Hospital of Guizhou Medical University, Guiyang, Guizhou China; 2 MP Biochemicals, Auckland, New Zealand; 3 Department of Pathology, Memorial Sloan Kettering Cancer Center, New York, USA; 4 Curandis, New York, USA

**Keywords:** Autoantigen, Autoantibody, Autoimmune liver disease, Hepatitis

## Abstract

**Background:**

Autoimmune diseases result from aberrant immune attacks by the body itself. It is mysterious how autoantigens, a large cohort of seemingly unconnected molecules expressed in different parts of the body, can induce similar autoimmune responses. We have previously found that dermatan sulfate (DS) can form complexes with molecules of apoptotic cells and stimulate autoreactive CD5+ B cells to produce autoantibodies. Hence, autoantigenic molecules share a unique biochemical property in their affinity to DS. This study sought to further test this uniform principle of autoantigenicity.

**Results:**

Proteomes were extracted from freshly collected mouse livers. They were loaded onto columns packed with DS-Sepharose resins. Proteins were eluted with step gradients of increasing salt strength. Proteins that bound to DS with weak, moderate, or strong affinity were eluted with 0.4, 0.6, and 1.0 M NaCl, respectively. After desalting, trypsin digestion, and gel electrophoresis, proteins were sequenced by mass spectrometry. To validate whether these proteins have been previously identified as autoantigens, an extensive literature search was conducted using the protein name or its alternative names as keywords. Of the 41 proteins identified from the strong DS-affinity fraction, 33 (80%) were verified autoantigens. Of the 46 proteins with moderate DS-affinity, 27 (59%) were verified autoantigens. Of the 125 proteins with weak DS-affinity, 44 (35%) were known autoantigens. Strikingly, these autoantigens fell into the classical autoantibody categories of autoimmune liver diseases: ANA (anti-nuclear autoantibodies), SMA (anti-smooth muscle autoantibodies), AMA (anti-mitochondrial autoantibodies), and LKM (liver-kidney microsomal autoantigens).

**Conclusions:**

This study of DS-affinity enrichment of liver proteins establishes a comprehensive autoantigen-ome for autoimmune liver diseases, yielding 104 verified and 108 potential autoantigens. The liver autoantigen-ome sheds light on the molecular origins of autoimmune liver diseases and further supports the notion of a unifying biochemical principle of autoantigenicity.

## Background

The etiology of autoimmune diseases in general has remained a biomedical mystery. It is not clear how and why some molecules or tissue components of the body become a self-target of the immune defense system, whereas most do not. In previous studies, we demonstrated that certain molecules from dying cells have affinity to dermatan sulfate (DS), and that these molecules can form macromolecular complexes with DS to co-stimulate autoreactive CD5+ B cells to secrete autoantibodies [1]. Furthermore, we demonstrated that molecules with affinity to DS have a high propensity to be autoantigens (autoAg) [2]. We proposed a uniform principle of autoantigenicity that explains how a vast variety of seemingly unrelated molecules can become autoantigenic by means of a shared biochemical property. In this study, we sought to test this principle and to define the repertoire of possible autoantigens, i.e., the autoantigen-ome, in autoimmune liver diseases.

Autoimmune diseases of the liver occur when the body’s own immune system attacks the liver [3–5]. These diseases have different clinical patterns with regard to degree of severity and clinical course, but they all share one important feature, i.e., the liver being the target of an aberrant autoimmune attack by autoantibodies and/or autoreactive cells. Autoimmune liver diseases are typically chronic conditions, and patients may experience persistent or recurrent autoimmune insults to the liver, often without overt symptoms. As the autoimmune attack persists, liver tissue scars and leads to hepatic fibrosis; and as fibrosis progresses to cirrhosis, liver function is compromised. Ultimately, end-stage liver disease and liver failure may ensue, requiring organ transplantation.

Among autoimmune diseases of the liver, autoimmune hepatitis (AIH) [3], primary biliary cirrhosis (PBC) [4], and primary sclerosing cholangitis (PSC) [5] are the most prominent. In AIH, the immune system attacks the hepatocytes and causes chronic inflammation of the liver. About 70% of AIH patients are female. In PBC, the autoimmune reaction is directed at small biliary ducts inside the liver. In PSC, autoimmunity targets the larger extrahepatic bile ducts. Characteristic morphological patterns are chronic inflammation and a hepatic pattern of injury with prominent plasma cells in AIH, destruction of small intrahepatic bile ducts and canals of Hering in PBC, and periductal fibrosis and inflammation of the larger bile ducts, often along with inflammatory bowel disease, in PSC. Although most liver autoimmune diseases fall into these three categories, overlaps and other syndromes also occur.

Autoimmune liver diseases are typically associated with several classes of autoantibodies, including ANA, AMA, anti-SMA/anti-F-actin, anti-LKM, and others [6, 7]. For AIH and PBC, testing for liver-related autoantibodies is a prerequisite for diagnosis. For PSC, autoantibodies are frequently present but their diagnostic value has not been established. When diagnosed at an early stage, autoimmune hepatitis can be controlled by daily doses of steroids and other medicines that suppress inflammation. However, these treatments only suppress or slow down the overactive immune system, but cannot cure the disease. Understanding the molecular origins of autoimmune liver diseases is therefore crucial to finding more effective therapies.

## Methods

### DS-Sepharose resin synthesis

DS-Sepharose resins were prepared by coupling dermatan sulfate (DS; Sigma-Aldrich) to EAH Sepharose 4B resins (GE Healthcare). Sepharose resins (20 ml) were washed with distilled water and 0.5 M NaCl and then mixed with 100 mg of DS dissolved in 10 ml of 0.1 M MES buffer (pH 5.0). N-ethyl-N-(3-dimethylaminopropyl) carbodiimide hydrochloride (Sigma-Aldrich) was added to a final concentration of 0.1 M. The reaction proceeded at 25 °C for 24 h with end-over-end rotation. After the first 60 min, the pH of the reaction mixture was readjusted to 5.0. After the coupling, the resins were washed three times, each time with a low pH buffer (0.1 M acetate, 0.5 M NaCl, pH 5.0) and a high pH buffer (0.1 M Tris, 0.5 M NaCl, pH 8.0). The washed DS-Sepharose resins were suspended in 10 mM phosphate buffer (pH 7.4) and packed into a C16/20 column (GE Healthcare). The column was equilibrated with 10 mM phosphate buffer before use.

### Mouse liver protein extraction

Livers were obtained from 5-month-old BALB/cJ female mice (Jackson Laboratory, Bar Harbor, Maine). The mouse tissue use was approved by the Institutional Animal Care and Use Committee (IACUC) of Brigham and Women’s Hospital (Boston, MA). All animal care was provided according to institutional, local, state, and federal regulations at the Brigham and Women’s Hospital research animal facility. A total of 20 mice were killed with CO_2_, their blood was removed through heart puncture, and their livers were collected immediately. Livers were cleaned by rinsing with phosphate buffered saline (PBS, pH 7.2) twice and then stored at 4 °C for 1 h, -20 °C for 2 h, and then -80 °C until further processing. Thawed livers were cut to small pieces and pressed through a cell strainer (Fisher Scientific). To remove red blood cells, the liver tissue was mixed with 10 ml of RBC (red blood cell) lysis buffer for 10 s. After centrifugation for 5 min, the supernatant was discarded. The liver tissue was mixed with 40 ml of RIPA lysis buffer (Sigma-Aldrich) and 4 tablets of protease inhibitor (cOmplete protease inhibitor cocktail, Sigma-Aldrich). The tissue mixture was sonicated for 10 min or until all tissue pieces appeared dissolved. The mixture was centrifuged at 13,300 rpm for 20 min, and the supernatant that contains total soluble liver proteins was collected. Protein concentration was measured by the RC DC protein assay (Bio-Rad).

### DS-affinity fractionation

Pilot affinity fractionations were performed in small centrifuge tubes. Aliquots of 2 ml resin were centrifuged to remove the storage buffer, and 0.5 ml of extracted liver proteins was added. The tube was mixed by rotating end-over-end at room temperature for 1 h to allow sufficient binding of proteins to DS-resin. The resin slush was centrifuged, and the supernatant containing unbound proteins was removed. The resins were washed 4 times with 1 ml of 0.2 M NaCl in 10 mM phosphate buffer to further remove unbound or poorly bound proteins. Proteins bound to DS with weak affinity were released from the resins by 0.4 M NaCl in PBS by rotating end-over-end at room temperature for 30 min, and the supernatant containing weak-binding proteins was recovered by centrifugation. The high-affinity proteins still bound to DS-resin were obtained by boiling the resins with 0.2 ml of SDS-PAGE sample buffer. Proteins in each fraction were assessed by 1D gel electrophoresis.

After the pilot assessment, fractionation of larger quantities of liver proteins was carried out by FPLC using a Biologic Duo-Flow System (Bio-Rad). Liver proteins were loaded into the DS-Sepharose column in 10 mM phosphate buffer at a rate of 1 mL/min. The column was washed with 20 mL of buffer A to remove unbound proteins. Proteins bound to DS resins with weak-to-strong affinity were eluted with a step-gradient of 0.2 M, 0.4 M, 0.6 M, and 1.0 M NaCl in 10 mM phosphate buffer, with each step being 40 ml. Elution was monitored by UV and conductivity detectors. All bound fractions were collected. Fractions were concentrated and desalted in Vivaspin centrifugal concentrators (MWCO 10 kDa, Sigma-Aldrich). Concentrated proteins were reconstituted in 10 mM phosphate buffer for further analysis.

### Protein sequencing by mass spectrometry

Fractionated proteins with different affinities to DS were separated on 1D SDS PAGE in 4–12% NuPAGE Novex Bis-Tris gels (Invitrogen). Based on protein band intensity, the protein lane containing proteins eluting at 0.4 M NaCl was cut into 3 sections, containing top, middle, and bottom bands. The lanes containing proteins eluting at 0.6 M and 1.0 M NaCl were each cut into 2 sections, containing top and bottom bands, respectively. Gel sections were transferred into 1-ml tubes, cut into tiny pieces, dehydrated with acetonitrile, and then dried in a speed-vac. Proteins in gel pieces were then rehydrated in 50 mM NH_4_HCO_3_ and digested with 12.5 ng/μl modified sequencing-grade trypsin (Promega) at 4 °C overnight.

Mass spectrometric sequencing was performed at the Taplin Biological Mass Spectrometry Facility (Harvard Medical School, Boston, USA). Tryptic peptides were separated on a nano-scale C18 HPLC capillary column and analyzed after electrospray ionization in an LTQ linear ion-trap mass spectrometer (Thermo Scientific). Peptide sequences and protein identities were assigned by matching protein or translated nucleotide databases with the measured fragmentation pattern using Sequest software. Peptides were required to be fully tryptic peptides with XCorr values of at least 1.5 (+ 1 ion), 1.5 (+ 2 ion), or 3.0 (+ 3 ion). All data were manually inspected. Only proteins with at least 2 peptide matches were considered confidently identified.

## Results

### Fractionation of liver proteins by DS-affinity

Proteins extracted from mouse livers were separated into 4 fractions according to their strength of binding to DS: no-, weak-, medium-, and strong-affinity. This was carried out by loading the liver proteins onto DS-Sepharose columns to allow binding to take place. Proteins that did not bind to DS resins were washed off the column with the 10 mM phosphate loading buffer, followed by washing with 0.2 M NaCl and 10 mM phosphate buffer. Afterwards, proteins that had remained bound to DS were sequentially eluted from the column with 0.4 M, 0.6 M, and 1.0 M NaCl, designating these as weak-, medium-, and strong-affinity fractions, respectively. Elution was monitored for presence of proteins, and individual chromatographic fractions containing proteins at each of the salt strengths were pooled, desalted, and concentrated. Protein content and protein size distribution of the fractions were assessed with 1D SDS PAGE gels.

The majority of liver tissue proteins not binding to DS were observed in the flow-through, and non-specific binding proteins were further washed with 0.2 M NaCl. As the elution proceeded, the amount of proteins eluting at increasing ionic strength became smaller and smaller. Proteins eluting last off the column with 1.0 M NaCl had the highest affinity to DS but were also the relatively least abundant. The flow-through and 0.2 M NaCl washed proteins were not further analyzed. Proteins eluting at 0.4 M, 0.6 M, and 1.0 M salt were sequenced by LC-MS/MS, yielding 168, 68, and 41 identified protein entities, respectively. Some of the proteins were redundantly identified in 2 or 3 fractions and, when redundancies were excluded, the number of different uniquely identified proteins in the 0.4 M, 0.6 M, or 1.0 M fraction was found to be 125, 46, and 41, respectively.

### Proteins with strong DS-affinity eluting at 1.0 M ionic strength

Proteins eluting off the DS-Sepharose column at 1.0 M NaCl were classified as having strong DS-affinity. From the 1.0 M elution, 41 proteins were identified by MS sequencing (Table 1). A literature search revealed that at least 33 (80.5%) of these have previously been identified as autoantigens for autoantibodies. Furthermore, these autoantigens were not randomly distributed in functional attribution but fell nicely into 5 classical categories of autoantibodies in autoimmune liver diseases: ANA (antinuclear autoantibodies), SMA (smooth muscle autoantibodies), AMA (anti-mitochondrial autoantibodies), LKM (liver-kidney microsomal autoantigens), and peroxisome (Table 1).

**Table 1:** Liver proteins with strong DS-affinity

^a^			ANA (antinuclear autoantigens)	Ref.
10	IPI00329998.3	H4	Histone H4	[8]
6	IPI00114642.4	Hist1h2bj	Histone H2B type 1-F/J/L	[9]
5	IPI00153400.2	H2afj	Histone H2A.J	[9]
5	IPI00111957.3	Hist1h2ba	Histone H2B type 1-A	[9]
4	IPI00137852.5	H2afy	Core histone macro-H2A.1	[10]
2	IPI00404590.1	H1f0	Histone H1.0	[11]
3	IPI00119220.1	Snrpd2	Small nuclear ribonucleoprotein Sm D2	[12]
2	IPI00114052.1	Snrpb	Small nuclear ribonucleoprotein-associated protein B	[13]
2	IPI00122350.4	Snrpa	U1 small nuclear ribonucleoprotein A	[14]
2	IPI00133955.1	Snrpe	Small nuclear ribonucleoprotein E	[15]
3	IPI00131988.1	Mrpl49	39S ribosomal protein L49, mitochondria	[16]
3	IPI00311236.1	Rpl7	60S ribosomal protein L7	[17]
2	IPI00122421.5	Rpl27	60S ribosomal protein L27	[18]
2	IPI00222549.6	Rpl30	60S ribosomal protein L30	[16]
3	IPI00124287.1	Pabpc1	Polyadenylate-binding protein	[19]
2	IPI00119959.1	Banf1	Barrier-to-autointegration factor	[20]
			SMA (anti-smooth muscle autoantigens) / Cytoskeleton	
6	IPI00753793.2	Spna2	Isoform 2 of Spectrin alpha chain	[21]
5	IPI00123181.4	Myh9	Myosin-9	[22]
2	IPI00109044.8	G15Rik	Myosin light chain, regulatory B-like	[23]
2	IPI00354819.5	Myl6	Isoform Smooth muscle of Myosin light chain 6	[24]
2	IPI00230435.1	Lmna	Isoform C2 of Lamin-A	[25]
			AMA (anti-mitochondrial autoantigens)	
13	IPI00319992.1	Hspa5	78 kDa glucose-regulated protein (Grp78)	[26]
13	IPI00114209.1	Glud1	Glutamate dehydrogenase 1, mitochondria	[27, 28]
11	IPI00131424.3	Cpt2	Carnitine O-palmitoyltransferase 2, mitochondria	
6	IPI00133903.1	Hspa9	Stress-70 protein, mitochondria (Grp75)	[29]
3	IPI00111908.8	Cps1	Carbamoyl-phosphate synthase [ammonia], mitochondria	
3	IPI00273146.1	Chdh	Choline dehydrogenase, mitochondria	
2	IPI00129577.1	Aifm1	Apoptosis-inducing factor 1 (Aif, Pdcd8, programmed cell death protein 8), mitochondria	[30]
			LKM (liver-kidney microsomal autoantigens)	
3	IPI00111936.1	Ugt1a9	UDP-glucuronosyltransferase 1-9 (bilirubin-specific)	[31]
3	IPI00112322.2	Ugt2b5	UDP glucuronosyltransferase 2 family, polypeptide B5	[32]
2	IPI00230113.5	Cyb5	Microsomal cytochrome b5	[33]
2	IPI00131771.3	Cox6c	Cytochrome c oxidase subunit 6c	
2	IPI00169666.3	Ugt2b34	UDP glucuronosyltransferase 2 family, polypeptide B3	[32]
2	IPI00230108.6	Pdia3	Protein disulfide-isomerase A	[34]
2	IPI00134746.5	Ass1	Argininosuccinate synthase	
2	IPI00387289.3	Ces3	Carboxylesterase	[35]
			Peroxisomal proteins	
3	IPI00112549.1	Acsl1	Long-chain-fatty-acid-CoA ligase	
2	IPI00312058.5	Cat	Catalase	[36, 37]
2	IPI00223367.5	Uox	Uricase	
2	IPI00125325.1	Decr2	Peroxisomal 2,4-dienoyl-CoA reductase	
2	IPI00125813.1	Dpp4	Dipeptidyl peptidase (CD26)	[38, 39]

^a^Columns left to right: Number of peptides identified for the protein by mass spectrometry; Protein ID; Gene name; Protein name; Reference (if any) reporting autoantibodies induced by the protein

Among the 41 proteins identified, 16 belong to the ANA family. They include 6 histones, 4 small nuclear ribonucleoproteins, 4 ribosomal proteins, and 2 others (polyadenylate-binding protein and barrier-to-autointegration factor). All of them are established ANA autoantigens (see references in Table 1). There are 5 proteins belonging to the SMA family, including spectrin alpha chain, myosin-9, myosin light chain, and lamin-A. All of them are known targets of autoantibodies (see references in Table 1). Among the AMA autoantigens, 7 were identified, with 4 having been verified as autoantigens, including Hspa5, Hspa9, glutamate dehydrogenase (Glud1), and apoptosis-inducing factor 1 (Aifm1). Autoantibodies to heat shock proteins (HSP) are widely found in autoimmune diseases as well as in numerous other diseases. Autoantibodies to HSP have been found in the circulation of various cancer patients, and are proposed as diagnostic and prognostic markers for various cancers such as breast cancer [40]. Autoantibodies to Glud1 have not been reported in humans but in mice and calves [27, 28]. Autoantibodies to Cpt2, Cps1, and Chdh could not be found in published reports. They could well represent autoantigens that have yet to be validated.

Autoantibodies to microsomal LKM antigens are associated with type 2 autoimmune hepatitis. Among proteins with high DS-affinity, 8 microsomal proteins were identified, including 3 of the UDG-glucuronosyltransferase family, 2 cytochromes, and 3 enzymes. Six of them (Ugt1a9, Ugt2b5, UgtCyb5, Cyb5, Pdia3, and Ces3) are known bona fide autoantigens (Table 1). Autoantibodies to Cox6c and Ass1 have not yet been described in literature. There are 5 proteins associated with the peroxisome in the 1.0 M elution fraction. Catalase and dipeptidyl peptidase (CD26, Dpp4) are reported autoantigens (Table 1). Autoantibodies to Acsl1, Uox, and Decr2 have not yet been reported.

### Proteins with moderate DS-affinity eluting at 0.6 M ionic strength

From fractions eluting at 0.6 M salt from DS-affinity columns, 68 proteins were identified by MS, but 22 of them were also present in the 1.0 M fraction. Therefore, 46 unique proteins were found (Table 2). Similarly to those with strong DS-affinity as described above, these proteins fell nicely into 5 categories of well-known autoantibodies: ANA, SMA, AMA, LKM, and peroxisome.

**Table 2:** Liver proteins with moderate DS-affinity

^a^			ANA (antinuclear autoantigens)	Ref.
8	IPI00230730.4	Hist2h3b	Histone H3.2	[41]
4	IPI00223713.5	Hist1h1c	Histone H1.2	[42]
2	IPI00459318.1	Hist1h2bp	Putative uncharacterized protein, histone	[9]
2	IPI00136632.3	H2afy3	Histone H2A member Y3	[43]
2	IPI00320149.2	H2afv	Histone H2A member V	[9]
6	IPI00308706.4	Rpl5	60S ribosomal protein L5	
5	IPI00127085.6	Rpl10a	60S ribosomal protein L10a	
3	IPI00555113.2	Rpl18	60S ribosomal protein L18	
3	IPI00138892.2	Uba52	Ubiquitin-60S ribosomal protein L40	
2	IPI00122598.3	EG382723	Similar to ribosomal protein L10	
5	IPI00339468.4	Dhx9	Isoform 2 of ATP-dependent RNA helicase, DNA helicase II	[44]
4	IPI00121596.3	Prpf8	Pre-mRNA-processing-splicing factor 8	
3	IPI00109764.2	Top1	DNA topoisomerase 1 (Scl-70)	[45]
2	IPI00322749.3	Snrpd1	Small nuclear ribonucleoprotein Sm D1	[46]
2	IPI00120162.1	Csnk2a1	Casein kinase II subunit alpha	[17]
			SMA (anti-smooth muscle autoantigens) / Cytoskeleton	
6	IPI00400300.1	Lmna	Isoform C of Lamin-A	[47]
4	IPI00113886.1	Lmnb2	Isoform B3 of Lamin-B	[48]
2	IPI00126191.5	Lmnb2	Isoform B2 of Lamin-B	[49]
2	IPI00113824.1	Hspg2	Basement membrane-specific heparan sulfate proteoglycan core protein	[50]
			AMA (anti-mitochondrial autoantigens)	
5	IPI00331555.2	Bckdha	Branched chain keto acid dehydrogenase E1, alpha polypeptide (mitochondrion matrix)	[51]
5	IPI00111877.1	Ssbp1	Single-stranded DNA-binding protein, mitochondria	
4	IPI00153144.3	Suox	Sulfite oxidase, mitochondria	[52]
3	IPI00133208.3	Hspa1l	Heat shock 70 kDa protein 1L	[53]
2	IPI00323357.3	Hspa8	Heat shock cognate 71 kDa protein	[54]
3	IPI00420718.4	Hmgcs2	Hydroxymethylglutaryl-CoA synthase, mitochondria	[27]
3	IPI00223092.5	Hadha	Trifunctional enzyme subunit alpha, mitochondria	
3	IPI00135651.1	Slc25a13	Calcium-binding mitochondrial carrier protein Aralar2	
3	IPI00111885.1	Uqcrc1	Cytochrome b-c1 complex subunit 1, mitochondria	[33]
3	IPI00132799.4	C1qbp	Complement component 1 q subcomponent binding protein	
2	IPI00127841.3	Slc25a5	ADP/ATP translocase (mitochondrion inner membrane)	
2	IPI00387379.1	Decr1	2,4-dienoyl-CoA reductase, mitochondria	
			LKM (liver-kidney microsomal autoantigens)	
6	IPI00117914.3	Arg1	Arginase-1	[55]
6	IPI00115679.1	Ganab	Isoform 2 of Neutral alpha-glucosidase A	[56]
4	IPI00621548.2	Por	NADPH-cytochrome P450 reductase	[57]
4	IPI00134691.3	Ugt1a1	UDP-glucuronosyltransferase 1-1	[58]
2	IPI00110556.1	Cyp2e1	Cytochrome P450 2E1	[59, 60]
2	IPI00321644.3	Cyp2d26	Cytochrome P450 2D26 (mouse) (LKM1 human)	[31]
			Peroxisomal proteins	
6	IPI00127558.3	Acox1	Peroxisomal acyl-coenzyme A oxidase	
6	IPI00127276.1	Ehhadh	Enoyl-Coenzyme A hydratase/3-hydroxyacyl CoA dehydrogenase (peroxisomal bifunctional enzyme)	[16]
2	IPI00331628.5	Hsd17b4	Peroxisomal multifunctional enzyme type (17 beta-hydroxysteroid dehydrogenase type 4)	[61]
			Miscellaneous	
4	IPI00115599.6	Hsd11b1	Corticosteroid 11-beta-dehydrogenase isozyme	
3	IPI00117705.1	Ddost	Dolichyl-diphosphooligosaccharide-protein glycosyltransferase 48 kDa subunit	
3	IPI00313236.3	Slc27a5	Bile acyl-CoA synthetase	
2	IPI00309035.2	Rpn1	Dolichyl-diphosphooligosaccharide-protein glycosyltransferase subunit 1 (ribophorin 1)	
2	IPI00127016.1	Hsd17b6	Hydroxysteroid 17-beta dehydrogenase	[62]
2	IPI00130985.1	Rdh7	Retinol dehydrogenase	

^a^Columns left to right: Number of peptides identified for the protein by mass spectrometry; Protein ID; Gene name; Protein name; Reference (if any) reporting autoantibodies induced by the protein

There are 15 proteins belonging to the ANA family, including 5 histones and 5 ribosomal proteins (Table 2). Aside from these, other interesting autoantigens were identified. DNA topisomerase 1, the classical Scl-70 autoantigen, was identified [45]. Casein kinase II was also identified [17]. Dxh9 (ATP-dependent RNA helicase and DNA helicase II) may resemble the classical Ku antigen [44]. Prpf8, a pre-mRNA-processing-splicing factor, has not been reported as an autoantigen.

There are 4 proteins belonging to the SMA family. Aside from 3 isoforms of lamin, autoantigen Hspg2 (basement membrane-specific heparan sulfate proteoglycan core protein) was found in the 0.6 M elution. Among the 12 proteins in the AMA family, autoantibodies to 6 have been reported, including Bckdha, Suox, Hspa1l, Hmgcs2, Uqcrc1, and Hspa8. Although autoantibodies to C1q have been widely studied, anti-C1qbp (complement C1q subunit binding protein) has not yet been reported. Ssbp1, Hadha, Scl25a5, or Decr1 were not found in the literature as autoantigens.

From the 0.6 M elution, all 6 proteins identified in the LKM family are reported autoantigens (see references in Table 2). The classical cytochrome P450 antigens including LKM1 were identified. Other autoantigens identified are UDP-glucuronosyltransferase 1, Arg1, and Ganab. In addition to the above classical categories of autoantigens, 6 proteins identified in the 0.6 M elution are miscellaneous. Except for Rdh7 being a reported autoantigen, Hsd11b1, Ddost, Slc27a5, Rpn1, and Hsd17b6 autoantibodies remain to be characterized.

### Proteins with weak DS-affinity eluting at 0.4 M ionic strength

From fractions eluting at 0.4 M salt, 168 proteins were initially identified. Among these, 18 were also found in both 0.6 M and 1.0 M elution, including H2bj, H2afj, H2afy, Snrpb, Myh9, Lmna, Hspa9, Hspa5, Cps1, Cpt2, Glud1, Ugt2b5, Pdia3, Ass1, Acsl1, Cat, Uox, and Aif. Among the rest, 3 (Sm D2, Spna2, and Ces) were found also in the 1.0 M elution but in not 0.6 M elution, and 22 proteins were found also in the 0.6 M but not in the 1.0 M elution. After excluding these redundancies, 125 proteins were found only in the 0.4 M elution (Table 3).

**Table 3:** Liver proteins with weak DS-affinity

^a^			ANA (antinuclear autoantigens)	Ref.
19	IPI00122011.2	Sf3b3	Isoform 1 of Splicing factor 3B subunit	[63]
14	IPI00420807.3	Sfrs1	Isoform 1 of Splicing factor, arginine/serine-rich	
2	IPI00153743.1	Sfrs7	Isoform 2 of Splicing factor, arginine/serine-rich	
4	IPI00123604.4	Rpsa	40S ribosomal protein SA	
3	IPI00469260.3	Eftud2	116 kDa U5 small nuclear ribonucleoprotein component	[64]
2	IPI00170008.1	Snrpa1	U2 small nuclear ribonucleoprotein A	[65]
2	IPI00114052.1	Snrpb	Small nuclear ribonucleoprotein-associated protein	[13]
2	IPI00119220.1	Snrpd2	Small nuclear ribonucleoprotein Sm D2	[66]
2	IPI00226073.2	Hnrnpf	Isoform 1 of Heterogeneous nuclear ribonucleoprotein F	
2	IPI00109860.3	Rbm8a	Isoform 2 of RNA-binding protein 8	
			SMA (anti-smooth muscle autoantigens)/Cytoskeleton	
19	IPI00123316.1	Tpm1	Isoform 1 of Tropomyosin alpha-1 chain	[67]
13	IPI00169707.2	Tpm3	Tropomyosin 3, gamm	[68]
9	IPI00230044.5	Tpm3	Isoform 2 of Tropomyosin alpha-3 chain	
2	IPI00421223.3	Tpm4	Tropomyosin alpha-4 chain	[69]
13	IPI00118899.1	Actn4	Alpha-actinin	[70]
9	IPI00113539.2	Fn1	Fibronectin	[71]
7	IPI00110850.1	Actb	Actin, cytoplasmic	[72]
6	IPI00110827.1	Acta1	Actin, alpha skeletal muscle	[72]
7	IPI00265380.4	Myh8	Myosin-8	[73]
3	IPI00129404.1	Myh6	Myosin-6	
3	IPI00114894.1	Myh11	Isoform 1 of Myosin-1	
5	IPI00230394.5	Lmnb1	Lamin-B	[74]
4	IPI00121892.9	Spnb2	Isoform 2 of Spectrin beta chain	[75]
3	IPI00227299.6	Vim	Vimentin	[69]
2	IPI00109061.1	Tubb2b	Tubulin beta-2B chain	[76]
			AMA (anti-mitochondrial autoantigens)	
52	IPI00129526.1	Hsp90b1	Endoplasmin	[26]
9	IPI00229080.7	Hsp90ab1	MCG1823	
4	IPI00330804.4	Hsp90aa1	Heat shock protein HSP 90-alpha	
39	IPI00136213.5	Sardh	Sarcosine dehydrogenase, mitochondrial	
31	IPI00468481.2	Atp5b	ATP synthase subunit beta, mitochondrial	[77]
21	IPI00130280.1	Atp5a1	ATP synthase subunit alpha, mitochondrial	
27	IPI00471246.2	Ivd	Isovaleryl-CoA dehydrogenase, mitochondrial	
17	IPI00134809.2	Dlst	Isoform 1 of Dihydrolipoyllysine-residue succinyltransferase component of 2-oxoglutarate dehydrogenase complex, mitochondrial	[78]
2	IPI00756386.1	Dhtkd1	Probable 2-oxoglutarate dehydrogenase E1 component DHKTD1, mitochondrial	
13	IPI00331564.2	Dld	Dihydrolipoyl dehydrogenase	
7	IPI00130535.1	Dbt	Lipoamide acyltransferase component of branched-chain alpha-keto acid dehydrogenase complex, mitochondrial	[78]
3	IPI00153660.4	Dlat	Dihydrolipoyllysine-residue acetyltransferase component of pyruvate dehydrogenase complex, mitochondrial	
13	IPI00387491.1	Aass	Alpha-aminoadipic semialdehyde synthase, mitochondrial	
13	IPI00468653.4	Pccb	Propionyl-CoA carboxylase beta chain, mitochondrial	
12	IPI00330523.1	Pcca	Propionyl-CoA carboxylase alpha chain, mitochondrial	
11	IPI00110843.3	Agmat	Agmatinase, mitochondrial	
9	IPI00114710.2	Pcx	Pyruvate carboxylase, mitochondrial isoform 2	
7	IPI00111218.1	Aldh2	Aldehyde dehydrogenase, mitochondrial	[27]
4	IPI00226430.2	Acaa2	3-Ketoacyl-CoA thiolase, mitochondrial	
3	IPI00119766.1	Rdh16	Retinol dehydrogenase 1	
3	IPI00405699.2	Aldh4a1	Delta-1-pyrroline-5-carboxylate dehydrogenase, mitochondrial	
3	IPI00121309.2	Ndufs3	NADH dehydrogenase [ubiquinone] iron-sulfur protein 3, mitochondrial	
3	IPI00753303.2	Npl22	Dihydrodipicolinate synthase-like, mitochondrial	
3	IPI00169862.1	Coq9	Ubiquinone biosynthesis protein COQ9, mitochondrial	
2	IPI00323592.2	Mdh2	Malate dehydrogenase, mitochondrial	
2	IPI00121105.2	Hadh	Hydroxyacyl-coenzyme A dehydrogenase, mitochondrial	
2	IPI00459725.2	Idh3a	Isoform 1 of Isocitrate dehydrogenase [NAD] subunit alpha, mitochondrial	[79]
2	IPI00133553.1	Mut	Methylmalonyl-CoA mutase, mitochondrial	
2	IPI00115607.3	Hadhb	Trifunctional enzyme subunit beta, mitochondrial	
2	IPI00130804.1	Ech1	Delta(3,5)-Delta(2,4)-dienoyl-CoA isomerase, mitochondrial	[80]
2	IPI00469195.2	Echdc2	Isoform 1 of Enoyl-CoA hydratase domain-containing protein 2, mitochondrial	
2	IPI00314909.2	Agxt	Alanine-glyoxylate aminotransferase	
2	IPI00226140.5	Maob	Amine oxidase [flavin-containing]	[81]
2	IPI00121440.4	Etfb	Electron transfer flavoprotein subunit beta	[82]
2	IPI00454008.1	Shmt2	Serine hydroxymethyltransferase	
			LKM (liver-kidney microsomal autoantigens)	
54	IPI00309073.2	Mttp	Microsomal triglyceride transfer protein	
33	IPI00153317.3	Aldh1l1	10-formyltetrahydrofolate dehydrogenase	
10	IPI00111936.1	Ugt1a9	UDP-glucuronosyltransferase 1-9	[31]
8	IPI00762897.2	Ugcgl1	UDP-glucose:glycoprotein glucosyltransferase	
3	IPI00127223.3	Ugt2b36	UDP-glucuronosyltransferase	
3	IPI00114778.1	Cyp2c37	Cytochrome P450 2C37	[83]
3	IPI00131176.1	mt-Co2	Cytochrome c oxidase subunit 2	
2	IPI00323908.1	Cyp2d10	Cytochrome P450 2D10	[83]
3	IPI00331322.3	Mgst1	Microsomal glutathione S-transferase	
9	IPI00115867.4	Ces1	Liver carboxylesterase	[35]
			Peroxisomal proteins	
6	IPI00331596.6	Pecr	Peroxisomal trans-2-enoyl-CoA reductase	
3	IPI00134870.3	Acox2	Peroxisomal acyl-coenzyme A oxidase	
5	IPI00110719.1	Pipox	Peroxisomal sarcosine oxidase	[84]
2	IPI00130924.1	Slc27a2	Very long-chain acyl-CoA synthetaselow-den	
2	IPI00121833.3	Acaa1a	Acaa1b 3-ketoacyl-CoA thiolase A, peroxisomal	
2	IPI00111235.2	Aldh3a2	Fatty aldehyde dehydrogenase variant	
			Apoptosis	
9	IPI00310240.4	Anxa6	Annexin A6 isoform	[85]
5	IPI00116498.1	Ywhaz	14-3-3 protein zeta/delta	[86]
5	IPI00118384.1	Ywhae	14-3-3 protein epsilon	[87]
5	IPI00230707.6	Ywhag	14-3-3 protein gamma	[87]
			Proteasome	
8	IPI00113845.1	Psmb1	Proteasome subunit beta type-1	[88]
4	IPI00119239.2	Psmb6	Proteasome subunit beta type-6	
3	IPI00116712.1	Psmb8	Proteasome subunit beta type-8	
2	IPI00128945.1	Psmb2	Proteasome subunit beta type-2	
2	IPI00129512.3	Psmb4	Proteasome subunit beta type-4	
2	IPI00136483.1	Psmb7	Proteasome subunit beta type-7	
5	IPI00331644.5	Psma3	Proteasome subunit alpha type-3	[88]
4	IPI00109122.1	Psma8	Proteasome subunit alpha type-7-like	
4	IPI00131845.1	Psma6	Proteasome subunit alpha type-6	
4	IPI00420745.7	Psma2	Proteasome subunit alpha type-2	
4	IPI00277001.4	Psma4	Proteasome subunit alpha type-4	[89]
			Miscellaneous	
68	IPI00123639.1	Calr	Calreticulin	[77]
67	IPI00271951.5	Pdia4	Protein disulfide isomerase A	
61	IPI00122815.3	P4hb	Protein disulfide-isomerase	[90]
17	IPI00222496.3	Pdia6	Putative uncharacterized protein	
2	IPI00163011.2	Txndc5	Thioredoxin domain-containing protein	
20	IPI00119618.1	Canx	Calnexin	[91]
18	IPI00622235.5	Vcp	Transitional endoplasmic reticulum ATPase	[92]
14	IPI00125514.1	Entpd5	Ectonucleoside triphosphate diphosphohydrolase 5	
11	IPI00475154.1	Rpn2	Dolichyl-diphosphooligosaccharide-protein glycosyltransferase subunit	
2	IPI00309035.2	Rpn1	Dolichyl-diphosphooligosaccharide--protein glycosyltransferase subunit	
11	IPI00112719.1	Alad	Delta-aminolevulinic acid dehydratase	
10	IPI00115680.1	Prkcsh	Isoform 1 of Glucosidase 2 subunit beta	
7	IPI00119063.2	Lrp1	Pro-low-density lipoprotein receptor-related protein (alpha-2-macroglobulin receptor, apolipoprotein E receptor)	
2	IPI00624663.3	Pzp	Alpha-2-macroglobulin	
7	IPI00135512.1	Cnpy2	Protein canopy homolog 2	
6	IPI00316314.1	Hacl1	2-hydroxyacyl-CoA lyase	
6	IPI00116254.1	Prdx4	Peroxiredoxin-4	[93]
6	IPI00125899.1	Ctnnb1	Catenin beta-1	
4	IPI00112963.1	Ctnna1	Catenin alpha-1	[94]
5	IPI00113869.1	Bsg	Isoform 2 of Basigin (M6, EMMPRIN, TCSF, CD147)	
4	IPI00126184.7	Gc	Vitamin D-binding protein	[95]
4	IPI00123342.4	Hyou1	Hypoxia up-regulated protein	
3	IPI00130950.1	Bhmt	Betaine--homocysteine S-methyltransferase	[96]
3	IPI00134058.3	Erp44	Endoplasmic reticulum resident protein ERp44	
3	IPI00387282.4	Aadac	Arylacetamide deacetylase	
3	IPI00122346.2	Ssr4	Signal sequence receptor, delta	
3	IPI00317740.5	Gnb2l1	Guanine nucleotide-binding protein subunit beta-2-like	
3	IPI00319973.3	Pgrmc1	Membrane-associated progesterone receptor component	
2	IPI00279218.1	Apeh	Isoform 2 of Acylamino-acid-releasing enzyme	
2	IPI00323624.3	C3	Isoform Long of Complement C3	[97]
2	IPI00116432.1	Fmo1	Dimethylaniline monooxygenase [N-oxide-forming]	
2	IPI00114044.1	Man2a1	Alpha-mannosidase	
2	IPI00312018.6	Mlec	Malectin	
2	IPI00115241.1	Mup4	Major urinary protein 4	

^a^Columns left to right: Number of peptides identified for the protein by mass spectrometry; Protein ID; Gene name; Protein name; Reference (if any) reporting autoantibodies induced by the protein

As shown in Table 3, these 125 proteins fell naturally into 8 categories: ANA (10 proteins), SMA (15 proteins), AMA (35 proteins), LKM (10 proteins), peroxisome (6 proteins), apoptosis (4 proteins), proteasome (11 proteins), and miscellaneous (34 proteins).

The ANA autoantigens with weak DS-affinity are primarily isoforms of splicing factors and small nuclear ribonucleoproteins. Although autoantibodies to splicing factors have been reported [63], the exact isoforms identified here have not been reported. Anti-smooth muscle autoantigens identified in the 0.4 M NaCl elution included various forms of tropomyosin, actinin, fibronectin, actin, myosin, lamin, spectrin, and tubulin. Among the 15 identified here, 12 are bona fide autoantigens. Among the 35 proteins associated with mitochondria, 32 are enzymes, with 8/32 being reported autoantigens. These enzymes are from diverse families, e.g., dehydrogenases, synthases, acyltransferases, or carboxylases.

Similar to those identified in the 1.0 M and 0.6 M factions, the LKM autoantigens included members of cytochrome and UDP-glucuronosyltransferase families. In addition, 3 unrelated proteins, Mttp, Aldh1l1, and Ces1 were identified. Ces1 is a verified autoantigen. Six enzymes associated with the peroxisome were identified. Thus far, only peroxisomal sarcosine oxidase has been described to be an autoantigen. In addition to the above autoantigen categories, additional proteins were found associated with apoptosis and the proteasome (Table 3). Annexin A6 and 14–3-3 proteins are reported autoantigens. Members of the proteasome have also been reported as autoantigens.

The remaining 34 proteins could not easily be classified into particular categories. The majority of them are not yet characterized as autoantigens. However, some are reported autoantigens, such as calreticulin, calnexin, catenin, protein disulfide-isomerase, peroxiredoxin 4, vitamin D-binding protein, and complement C3 (Table 3).

## Discussion

Under normal physiologic conditions, the immune system is designed to protect from infection and disease through intricate mechanisms that distinguish self from non-self. It is a mystery why and how the immune system is mistakenly triggered to attack the body’s self. Autoimmune responses are causally linked to autoantibodies, autoreactive cells, or both. Despite advances in our understanding of the many facets of autoimmunity, the underlying molecular and cellular mechanisms that trigger autoimmunity remain largely unknown.

We are intrigued by the question why and how a vast number of diverse, seemingly functionally disconnected proteins in different parts of the body and with diverse structures and biological functions can all induce a converging autoimmune response, i.e., the production of autoantibodies by autoreactive B cells. Based on our previous studies [1, 2], we concluded that autoantigens share a common biochemical property in their binding affinity to dermatan sulfate (DS), also called chondroitin sulfate B, a glycosaminoglycan-type mucopolysaccharide found mostly in skin but also in blood vessels, heart valves, tendons, lungs, and other tissues. DS can directly bind molecules released from dying cells or other sources and form macromolecular DS-autoantigen complexes, and such complexes, in turn, can stimulate autoreactive B cells through simultaneous engagement of multiple signaling molecules on the B cell surface to induce an activated B cell response. To further characterize our proposed “unifying principle of autoantigenicity” based on DS-affinity as a shared physicochemical property of autoantigens, we tested whether we could identify autoantigens from a specific parenchymal organ, and whether autoantigens showed preferential intrinsic biochemical propensity for high DS-affinity.

Autoimmune liver diseases result from the immune system mistakenly attacking hepatocytes or cholangiocytes in the liver [3–5]. Patients with these chronic conditions are usually initially rather asymptomatic, and autoantibody serology tests are often necessary to clarify the diagnosis [6, 7]. For example, while routine blood tests for liver enzymes can reveal patterns of hepatitis, further autoantibody tests are needed to diagnose autoimmune hepatitis. Autoantibody tests also help distinguish autoimmune hepatitis from other liver diseases, such as viral hepatitis or metabolic diseases such as Wilson disease.

Common autoimmune liver diseases include autoimmune hepatitis (AIH) [3], primary biliary cirrhosis (PBC) [4], and primary sclerosing cholangitis (PSC) [5]. An autoimmune liver disease panel (a series of tests that detect autoantibodies to common autoantigens associated with these diseases) include anti-liver-kidney microsomal antibodies (LKM), anti-mitochondrial antibodies (AMA), anti-nuclear antibodies (ANA), and anti-smooth muscle antibodies (SMA). AIH is further classified to two types, type I is defined by positive ANA and SMA, whereas type 2 is associated with anti-KLM autoantibodies. ANA occur in a wide variety of systemic autoimmune diseases, such as systemic lupus erythematosus, rheumatoid arthritis, Sjögren syndrome, and systemic sclerosis. Lupus hepatitis is regarded as a distinct manifestation of SLE [98]. The identification of 41 confirmed or putative ANA autoantigens from liver tissue uncovered by our study may perhaps explain the overlap autoantibody profile and clinical manifestations between lupus and AIH. AMA are hallmark diagnostic markers for PBC. In PBC, the targets are small bile ducts, but the prototypic serologic response is the production of a multilineage immune response to mitochondrial autoantigens. AMA are detected in 90–95% of PBC patients, although their presence is extremely low in the general population (varying between 0.16 and 1%) [99]. More than 60 autoantibodies have been detected in patients with PBC [99]. In our current study, we identified 54 verified and putative autoantigens associated with mitochondria.

Based on all of our observation so far, we find that autoantigens with the strongest DS affinity are typically DNA- and RNA-binding proteins. Other autoantigens largely display moderate to weak DS affinity. However, it should be noted that our definition of DS binding strength is arbitrary, with DS-autoAg complexes dissociable at 1.0, 0.6, and 0.4 M ionic strength defined as strongly, moderately, and weakly binding, respectively. All of these DS-binding proteins would be expected to remain in complexed forms with DS under physiologic conditions. For example, cytochrome P450 2D6 (CYP2D6) is the major autoantigen of LKM1 autoantibodies [100], but its mouse homologues (Cyp2d26 and Cyp2d10) were found to possess only moderate to weak DS affinity (Tables 2 and 3). As another example, PDC-E2 is a major autoantigen in PBC patients, but several components of the PDC (pyruvate dehydrogenase complex) were only identified in the weak but not the strong DS affinity fraction of this study (Table 3). Hence, these results suggest that proteins only need to exhibit some (sufficient) DS affinity to become potentially autoantigenic. It is also possible that in toto weakly DS-binding proteins may contain fragment epitopes with strong DS affinity, and such epitopes could determine the autoantigenicity of the protein.

The liver is the largest internal organ, the largest gland of the human body, and also the largest reservoir of human proteins. The liver serves hundreds of physiological functions, including removal of toxic substance, storage of glycogen, decomposition of red blood cells, production of bile and hormones, and synthesis of plasma proteins. Transcriptome analysis shows that 59% (*n* = 11,553) of all human proteins (*n* = 19,613) are expressed in the liver (The Human Protein Atlas). It should be noted that our DS-affinity approach provided a significant enrichment of liver protein autoantigens, yielding only a little over 200 proteins (i.e., around 1% of the total human proteome) as bona fide verified or potential autoantigens.

## Conclusions

**Fig. 1:**
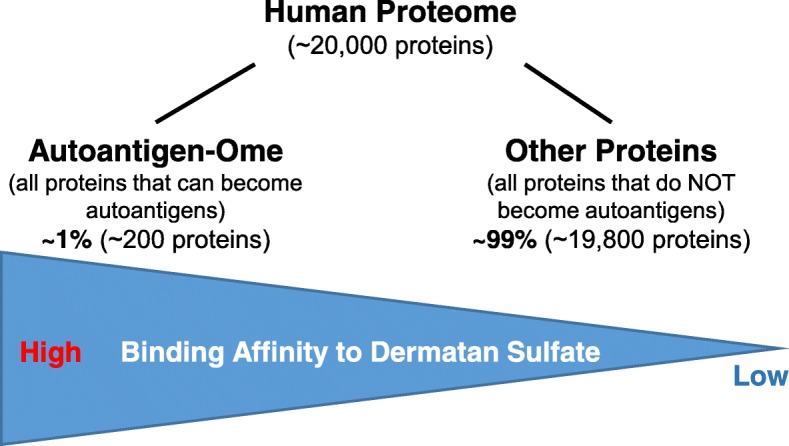
Model illustrating our hypothesis that binding affinity to dermatan sulfate is a distinct biochemical property characterizing (a small subset of) human proteins (estimated at ~ 1%) that can become human autoantigens, whereas the majority of proteins (estimated at ~ 99%) that lack binding affinity have a much lower propensity to become targets of autoimmunity

Our study of DS-affinity enrichment of the liver proteome produced a comprehensive autoantigen-ome that includes 104 bona fide autoantigens and 108 potential autoantigens for autoimmune liver diseases. These autoantigens fell into the classical categories of autoantibodies for autoimmune liver diseases. Our study provides further support to a model in which DS-affinity is a distinct biochemical property of proteins that can become autoantigens, whereas proteins that lack DS-affinity have a much lower propensity to be targets of autoimmunity (Fig. 1). These results may help in the further characterization of autoantigenic molecules and thus point to new innovative directions in autoimmunity research.

## Data Availability

All data generated or analyzed during this study are included in this published article.

## Abbreviations

AIH: Autoimmune hepatitis
AMA: Anti-mitochondrial autoantibodies
DS: Dermatan sulfate
LKM: Liver-kidney microsomal autoantigens
PBC: Primary biliary cirrhosis
PSC: Primary sclerosing cholangitis
SMA: Smooth muscle autoantibodies
